# *In Vitro* generation of Panton-Valentine leukocidin (PVL) in clinical *Methicillin-Resistant Staphylococcus aureus* (MRSA) and its correlation with PVL variant, clonal complex, infection type

**DOI:** 10.1038/s41598-018-26034-y

**Published:** 2018-05-16

**Authors:** Chuanling Zhang, Yuanyu Guo, Xu Chu

**Affiliations:** Department of Clinical Laboratory, Zhejiang Xiaoshan Hospital, 728 Yucai Rd., Hangzhou, Zhejiang Province People’s Republic of China

## Abstract

The amount of Panton-Valentine leukocidin (PVL) is diverse among *Staphylococcus aureus* isolates from different geographical regions, and its significance in some infections is disputed. However, data concerning this information in China are limited. Fifty-one *lukSF-PV*^+^ methicillin-resistant *Staphylococcus aureus* (MRSA) isolates gathered from varying infections were used for PVL production using enzyme-linked immunosorbent assay, and the quantity was analyzed in correlation with PVL isoform, genetic background of the isolate, and disease category. All isolates generated PVL with a range of 0.43–360.87 μg/mL, of which 56.9% isolates (29/51) generated 51–200 μg/mL of PVL; 11.8% (6/51) yielded PVL more than 200 μg/mL, and the rest (31.4%, 16/51) produced PVL of ≤50 μg/mL. The amount of PVL was not related to its variant and infection type, although isolates from skin and soft tissue infection had relatively high mean and median. Clonal complex (CC) 22 isolates might be the producer of relatively high concentrations of PVL; however, the difference among CCs was not analyzed due to a small number of CC isolates. The relevance of PVL production with the infection type, toxin isoform, and genetic characteristic of isolates may vary by clone type and also needs to be further evaluated using a large sample size and best concentration on *in vivo* environment.

## Introduction

Methicillin-resistant *Staphylococcus aureus* (MRSA) represents a major cause of infections in both the hospital and the community. The virulence of this pathogen partially relies on extracellular molecules. Panton-Valentine leukocidin (PVL, composed of *LukS-PV* and *LukF-PV*), a pore-forming toxin causing leukocytolysis and tissue necrosis, is one of these virulence factors that may have a significant influence in some serious *Staphylococcus aureus* (*S. aureus*) infections, such as severe skin and soft tissue infection, necrotizing pneumonia, and necrotizing fasciitis^1,2^. To date, PVL-producing MRSA isolates have emerged throughout the world^2–10^, and pose a huge threat to the health of patients. In Britain and France, therapeutic regimens may be regulated based on the existence or the absence of *S. aureus* PVL toxin^11^. The toxin-suppressing antibiotics, such as clindamycin, linezolid, and rifampin, are suggested for treating patients with serious PVL-expressing isolate infections^11^.

Previous reports have illustrated sequence variations within the *lukSF-PV* genes^8,12–15^. Molecular modeling indicates that a single amino acid replacement at site 176 [histidine (His) to arginine (Arg), namely H isoform changing into R isoform] may increase the leukotoxicity of PVL^8^. However, no evidence supports this assertion in the laboratory and clinical studies^13,16^. Genestier *et al*.^17^ reported that the role of PVL depends on the amount of toxin generated by *S*. *aureus*. Moreover, the only existence of a virulence gene does not imply that the toxin will be transcribed and/or translated and, if it is transcribed and/or translated, the toxin yield can be significantly different among isolates^6^. Therefore, this study detected the *in vitro* PVL production in clinical *lukSF-PV* positive (*lukSF-PV*^+^) MRSA isolates and explored whether the quantitative generation of PVL is correlated with specific isoform. In addition, the study also further analyzed the correlation of PVL production with clonal complexes (CCs) of isolates and type of infection.

## Results

### Clinical features

Fifty-one *lukSF-PV*^+^ MRSA isolates were isolated from 31 adult patients (including 14 young and 17 middle-aged and elderly patients), 1 adolescent, and 19 pediatrics (containing 9 children aged <1 year and 5 neonates aged <28 days). Of the 51 isolates, 19 were related to skin and soft tissue infections (SSTI), 16 to pneumonia, 5 to surgical site infections, and 11 to other infections (1 bacteremia, 1 urinary tract infection, 1 acute bacterial tracheitis, 4 tympanitis, and 4 conjunctivitis). According to the case notes, 22 (43.1%) and 29 (56.9%) MRSA were established as hospital-associated (HA) isolate and community-associated (CA) isolate, respectively (Table 1). Of 34 patients aged ≤44 years, 27 (79.4%) had CA infections. However, among 17 patients aged ≥45 years, only 2 (11.8%, 2/17) had CA infections (Table 1, ages of patients not shown).

**Table 1:** Characteristics of 51 *lukSF-PV*^+^ MRSA isolates

Infection type, isolate (*n*)	CA/HA	Molecular characteristic				
		Isoform of PVL	SCC*mec* type	PFGE type	MLST type	CC
Pneumonia (16)						
PVL74	CA	H2	III	A1	ST59	CC59
PVL130	HA	H1	III	F2	ST59	CC59
PVL161	CA	H2	NT	K	ST188	CC1
PVL211	HA	H1	III	E1	ST22	CC22
PVL212	HA	H2	IVa	G1	ST59	CC59
PVL214	HA	H2	III	E1	ST22	CC22
PVL217	HA	H2	III	G3	ST59	CC59
PVL219	HA	H2	III	D2	ST338	CC59
PVL220	HA	H2	III	D3	ST338	CC59
PVL222	HA	H2	II	M	ST149	CC5
PVL226	HA	H2	IVa	G2	ST59	CC59
PVL239	HA	H2	III	E2	ST22	CC22
PVL244	HA	H2	NT	W	ST88	CC88
PVL254	HA	H2	III	B1	ST59	CC59
PVL255	HA	H2	II	P	ST149	CC5
PVL256	CA	H1	III	B1	ST59	CC59
Skin and soft tissue infection (19)						
PVL8	CA	H2	III	B3	ST59	CC59
PVL16	CA	H2	III	H1	ST59	CC59
PVL34	CA	H2	IVa	S	ST59	CC59
PVL51	CA	H2	III	B3	ST59	CC59
PVL60	CA	H2	IVa	H2	ST59	CC59
PVL65	CA	H2	IVa	T	ST59	CC59
PVL79	CA	H2	IVa	A3	ST59	CC59
PVL104	CA	R2	NT	X	ST88	CC88
PVL108	CA	H2	III	F1	ST59	CC59
PVL145	CA	H2	III	F1	ST59	CC59
PVL148	HA	H2	IVb	O	ST9	CC9
PVL150	CA	R2	V	J	ST25	CC5
PVL170	HA	H2	III	A2	ST59	CC59
PVL195	CA	H2	III	N	ST59	CC59
PVL203	CA	H1	IVa	Z	ST1	CC1
PVL205	HA	H2	III	E1	ST22	CC22
PVL209	HA	H1	IVa	C1	ST59	CC59
PVL210	CA	H2	III	C3	ST59	CC59
PVL233	CA	H2	III	Q	ST30	CC30
Surgical site infection (5)						
PVL7	CA	H2	V	V	ST1	CC1
PVL82	HA	H2	III	A1	ST59	CC59
PVL236	HA	H2	III	C4	ST59	CC59
PVL237	CA	H2	III	C2	ST59	CC59
PVL242	CA	H1	III	D1	ST338	CC59
Other infections (11)						
PVL40	CA	R1	IVa	R	ST59	CC59
PVL69	HA	H2	III	L	ST217	CC22
PVL186	CA	H2	IVa	A4	ST59	CC59
PVL202	CA	H2	IVa	Y	ST88	CC88
PVL204	CA	H2	III	C3	ST59	CC59
PVL206	CA	R2	IVa	U	ST25	CC5
PVL218	HA	R2	IVa	I1	ST59	CC59
PVL221	CA	H2	III	D1	ST338	CC59
PVL238	CA	H2	IVa	I2	ST59	CC59
PVL246	CA	H2	IVa	D4	ST338	CC59
PVL253	HA	H2	III	B2	ST59	CC59

CA, Community-acquired isolate; HA, hospital-acquired isolate; MLST, multilocus sequence typing; NT, nontypeable; PFGE, pulsed-field gelelectrophoresis; SCCmec, staphyloccoccal cassette chromosome mec element; ST, sequence type.

### *In vitro* production of PVL

**Figure 1:**
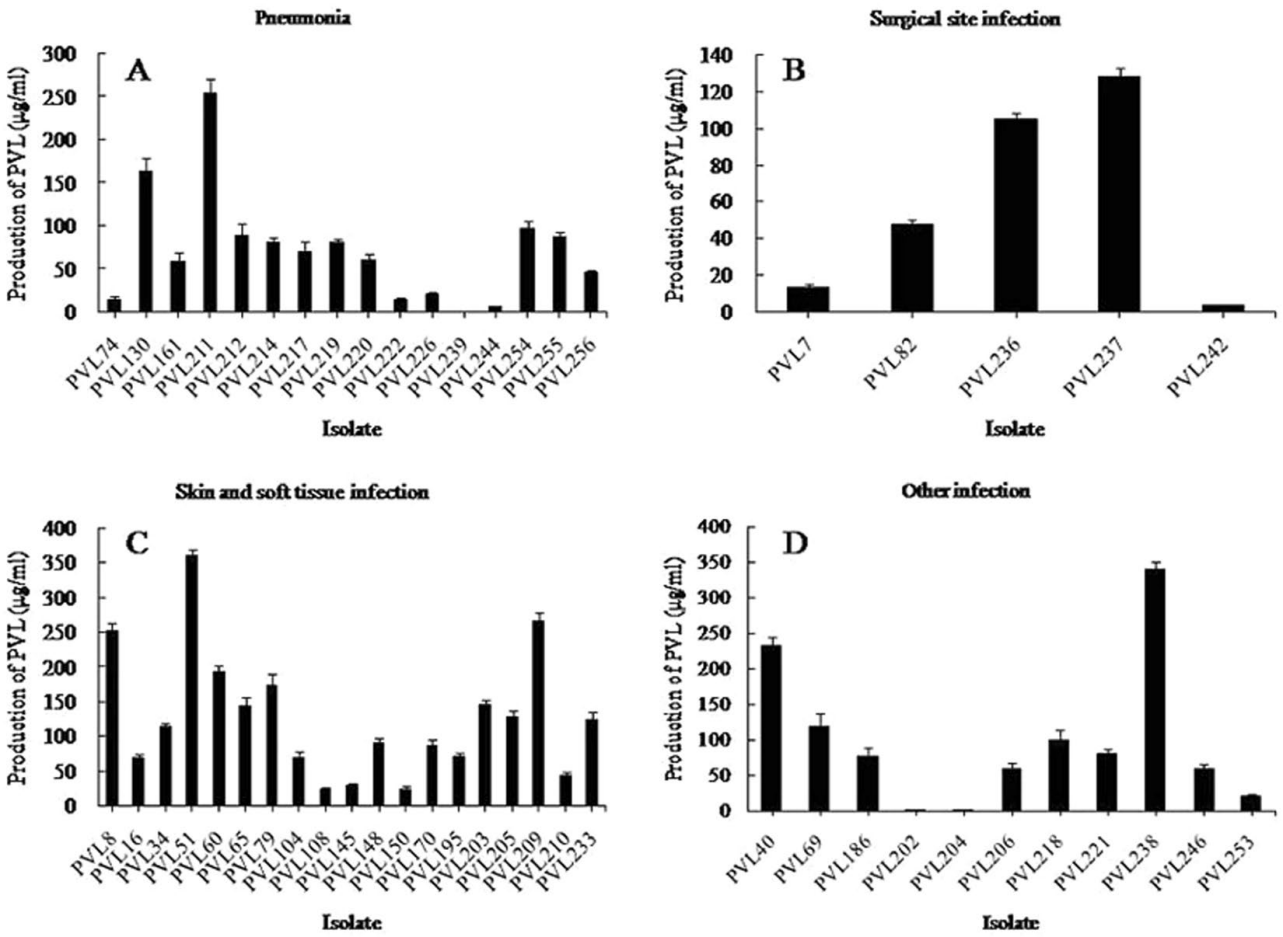
PVL yield of 51 clinical *lukSF-PV*^+^ MRSA isolates in a given infection classification. Isolates were grouped on the basis of the clinical diagnosis, and PVL generation was examined using ELISA. Error bars signify SDs of the results from three experiments for each isolate.

Twenty-nine (56.9%, 29/51) isolates generated PVL between 50 and 200 μg/mL, and 6 isolates had elevated PVL between 201 and 400 μg/mL; the remaining isolates (31.4%, 16/51) produced PVL less than 50 μg/mL. Pneumonia-related isolates yielded a mean [±standard deviation (SD)] of 72.11 ± 64.58 μg/mL and a median of 66.27 μg/mL PVL (Table 2). This group contained one isolate (isolate PVL239) for which extremely low PVL generation (0.94 μg/mL) was measurable (Fig. 1A). SSTI-associated isolates produced an average of 126.58 ± 90.67 μg/mL and a median of 113.63 μg/mL PVL (Table 2), among which one isolate (isolate PVL51) was the highest producer (360.87 μg/mL) (Fig. 1C). For the other infection-related isolates, an average PVL level of 98.34 ± 102.79 μg/mL and a median of 75.79 μg/mL were found (Table 2). This group included one isolate (isolate PVL202), which was the lowest producer (0.43 μg/mL) (Fig. 1D). Surgical site infection–associated isolates generated the lowest PVL quantity (a mean of 59.55 ± 55.11 μg/mL and a median of 47.72 μg/mL) (Table 2 and Fig. 1B). Although the isolates from SSTI had relatively high values, the results from the Kruskal–Wallis test displayed no significant differences in PVL yields among the four different disease groups (*P* = 0.151).

**Table 2:** ELISA data on *in vitro* PVL production among clinical isolates of *lukSF-PV*^+^ MRSA (*n* = 51).

Group	Number of isolates (*n*)	PVL range (μg/mL)	PVL median (μg/mL)	PVL mean ± SD (μg/mL)	*P* value
Clonal complex (CC)					
Isolates belonging to CC59	34	0.94**−**360.87	80.75	107.67 ± 91.92	NA
Isolates belonging to CC22	5	0.94**−**254.62	118.51	116.88 ± 75.98	NA
Isolates belonging to CC88	3	0.43**−**68.47	6.53	25.14 ± 37.64	NA
Isolates belonging to CC1	3	13.65**−**145.32	59.91	72.96 ± 66.80	NA
Isolates belonging to CC5	4	14.67**−**88.38	40.85	46.18 ± 33.72	NA
Isolates belonging to CC9	1				NA
Isolates belonging to CC30	1				NA
Type of infection					
Isolates from SSTI	19	23.31**−**360.87	113.63	126.58 ± 90.67	0.151
Isolates from pneumonia	16	0.94**−**254.62	66.27	72.11 ± 64.58	
Isolates from surgical site infection	5	3.48**−**128.27	47.72	59.55 ± 55.11	
Isolates from other infection	11	0.43**−**339.52	75.79	98.34 ± 102.79	
CA isolates	29	0.43**−**360.87	68.47	105.2 ± 96.6	0.754
HA isolates	22	0.94**−**265.80	87.58	86.8 ± 68.8	
Isoform of PVL					
H isoform isolates	46	0.43**−**360.87	80.75	96.91 ± 86.41	0.975
R isoform isolates	5	23.82**−**231.74	68.47	96.09 ± 80.40	

CA, Community-associated infection; HA, hospital-associated infection; NA, not applicable for the Kruska–Wallis rank-sum test due to a relatively small number of isolates; PVL, Panton–Valentine leukocidin; SD, standard deviation; SSTI, skin and soft tissue infection.

Among all CCs, the CC22 isolates produced the highest level of PLV (a mean of 116.88 ± 75.98 μg/mL and a median of 118.51 μg/mL), followed by CC59, CC1, CC5, and CC88 isolates (Table 2). Although the PVL levels of isolates with CC88 and CC5 were far lower than those of CC22 and CC59 isolates, the statistical analysis was not performed to analyze the significance of the difference because of the small number of isolates of most CCs (Table 2). In addition, this study also analyzed the correlation of PVL yields of isolates with CA and HA infections, and PVL H and R isoforms. Unfortunately, no statistical differences were found among them (Table 2).

## Discussion

Among *lukSF-PV*^+^ MRSA, 66.7% (34/51) were isolated from young and pediatric patients (≤44 years old), which included 93.1% (27/29) CA MRSA. This was consistent with the reports that CA MRSA mostly affects young individuals and children, and usually presents PVL^18^.

Previous reports showed that the *lukSF-PV* genes were a frequent genetic marker of CA MRSA isolates with SCC*mec*IV or SCC*mec*V^19,20^. Nevertheless, some studies have reported the *lukSF-PV*^+^ HA MRSA strains^21–25^. This study also found that 43.1% (22/51) *lukSF-PV*^+^ MRSA were HA MRSA strains (Table 1). The prevalence of *lukSF-PV*^+^ MRSA clonal lineages is diverse in different parts of the earth. For example, ST8 (CC8)-SCC*mec*IV (USA300) clone predominates in USA; ST59 (CC59)-SCC*mec*V_T_ clone circulates in Asia, ST30 (CC30)-SCC*mec*IV clone is prevalent in New Zealand, ST93 (CC93)-SCC*mec*IV clones are found mainly in Australia, ST80 (CC80)-SCC*mec*IV is the most frequent clone in Europe and the Middle East, ST88 (CC88)-SCC*mec*IV clone is identified mainly in Africa, and ST22 (CC22)-SCC*mec*IV and ST772 (CC1)-SCC*mec*V clones emerge mainly in India^18^. In the present study, ST59 (CC59)-SCC*mec*III was the predominant clone (35.3%, 18/51) of *lukSF-PV*^+^ MRSA isolates, followed by ST59 (CC59)-SCC*mec*IV (IVa/IVb, 25.5%, 13/51). However, for the former, 61.1% (11/18) of isolates were found to be CA MRSA; for the latter, 38.5% (5/13) of isolates were detected as HA MRSA. Historically, SCC*mec*III was considered as one of the epidemiological markers of the main HA MRSA clones, and SCC*mec*IV was often found in CA MRSA isolates^24^. However, contrary to these views, SCC*mec*III and SCC*mec*IV were found in both HA and CA *lukSF-PV*^+^ MRSA isolates in this study. This indicated that the *lukSF-PV*^+^ MRSA isolates carrying SCC*mec*III or SCC*mec*IV could disseminate between the hospital and the community in the region studied. The PFGE types of some isolates with SCC*mec*III supported the possibility of the transmission of HA strains of SCC*mec*III into the community (Table 1). In addition, the reports by Song *et al*.^26^ and Yao *et al*.^24^ also described this spread of HA and CA MRSA isolates from Taiwan, Hong Kong, Korea, Vietnam, and Wenzhou city of mainland China. Geng *et al*.^27^ reported the existence of ST338 (CC59) CA MRSA isolate in Taiwan and the southern region of China. In the present study, five *lukSF-PV*^+^ MRSA isolates were also identified to be ST338 type, including three CA isolates and two HA isolates. The investigation results of Song *et al*.^26^ showed that ST30 (CC30) was predominant in Asian areas. However, only one HA isolate was ST30 in this study. Additionally, ST22 (CC22) and ST88 (CC88), predominating in India^28,29^ and Africa^3^, respectively, were also found in the isolates in this study. However, the ST22 isolates were SCC*mec*III type, and not SCC*mec*IV detected in India. Although ST8 (CC8) and ST80 (CC80) prevailing in the USA and Europe, respectively, have been described in CA MRSA isolates from Japan and Korea (ST8), and Singapore and Malaysia (ST80), both MRSA clones are apparently scarce in Asian regions^26^ and were not found in the isolates in this study (Table 1).

According to the reports of Dumitrescu *et al*.^12^, O’Hara *et al*.^8^, and Tong *et al*.^14^, the R isoform of PVL was found in CC8 (mainly USA300 strains), CC1, and CC93 isolates, which were mainly from the USA and Australia; while the H isoform was mainly observed in non-USA isolates belonging mainly to CC121, CC30, and CC22. A few isolates belonging to CC1, CC5, CC6, CC25, CC59, and CC88 also carried the H isoform. Table 1 shows that the H isoform was harbored by 90.2% (46/51) of *lukSF-PV*^+^ MRSA isolates belonging primarily to CC59 (69.6%, 32/46), followed by CC22 (10.9%, 5/46), CC88 (6.5%, 3/46), CC5 (6.5%, 3/46), CC1 (6.5%, 3/46), CC9 (2.2%, 1/46), and CC30 (2.2%, 1/46); however, the R isoform was found only in five isolates belonging to CC59, CC88, and CC5, which were different from those (CC8, CC1, and CC93) described by the previous studies^8,12,14^.

PVL can lead to concentration-dependent necrosis and apoptosis of human polymorphonuclear neutrophils and lysis of human monocytes and macrophages^12^, but whether PVL is pathogenic or a marker of specific infection is controversial. Contradictory data may be associated with the quantity of PVL generated by individual isolates. Up to now, the number of *S. aureus* isolates producing PVL is mainly determined using polymerase chain reaction (PCR) for *lukSF-PV* genes or quantitative reverse transcription–PCR for *lukSF-PV* mRNA levels. However, the findings of these methods may not relate to the actual yield of PVL. Therefore, the present study directly detected the PVL toxin production using enzyme-linked immunosorbent assay (ELISA). This study showed that all the *lukSF-PV*^+^ isolates produced PVL *in vitro*, and the extent of the toxin expression varied among isolates (range from 0.43 to 360.87 μg/mL), even within specific genetic contexts (Table 2).

Undoubtedly, the presence of the genes encoding PVL does not necessarily imply that the toxin is produced in the setting of human infection. While this is true, it is important to note that neither does *in vitro* production. Additionally, the production of toxin is varied from culture media. Historical documents have reported the inter-isolate variability for ST8, ST80, ST93, and ST121 isolates^6,11,30^. The same phenomenon was reported for the STs detected in this study. The data showed that two ST59 CA isolates (PVL51 and PVL238) were the strongest producers of PVL (360.87 μg/mL and 339.52 μg/mL, respectively); and three ST59 (PVL8, PVL40, and PVL209) and one ST22 (PVL211) isolates produced 231.74–265.80 μg/mL of PVL (Table 1 and Fig. 1). However, two extremely low producers of PVL also belonged to ST59 and ST22 clones (PVL202 and PVL204, 0.94 μg/mL each) (Table 1 and Fig. 1). The amounts of PVL yielded by other MRSA isolates (including the remaining ST59 and ST22 isolates) varied between 0.43 and 192.96 μg/mL. The cause of the difference in PVL expression is not clear. Also, the correlation of PVL yield with the genetic background of isolates warrants further investigation. The PVL concentration of 0.33 μg/mL evoked the apoptosis of human granulocytes, and the injection of 0.3 μg/mL of toxin into the intracutaneous tissue of rabbits caused local inflammation and necrosis^11^. According to the *in vitro* PVL production in this study (≥0.43 μg/mL), all *lukSF-PV*^+^ isolates likely had the ability to cause an inflammatory response during infections. Shallcross *et al*.^31^ reported that PVL-producing clinical isolates had a link with SSTIs. In the present study, the mean value and median of PVL production of SSTI isolates were relatively high compared with those of isolates from pneumonia, surgical site infections, and other infections. However, the significant difference in the toxin levels was not found among the four groups (Table 2). This result was not consistent with the conclusion that PVL performed a more significant role in *S. aureus* SSTIs^31^. In addition, the isolates from invasive infections (such as pneumonia) were even shown to yield low mean and median of PVL (Table 2). The possible explanation was that the clinical outcome was correlated with host factors, or with the existence of other toxins^30^.

For PVL variants, the H and R isoform isolates generated similar mean concentrations and medians of PVL, and the difference between the two groups was not significant (*P* = 0.975) (Table 2). This result suggested that point mutations in the open reading frame of *lukSF-PV* genes might have no effect on the toxin production.

A previous study showed that CA MRSA infection was responsible for rapidly progressive and lethal diseases such as necrotizing pneumonia, severe sepsis, and necrotizing fasciitis. Also, some data sustained the opinion that PVL was accountable at least partially for the enhanced virulence of CA MRSA^32^. In this study, the CA and HA isolates yielded similar levels of PVL (*P* = 0.754) (Table 2). This indicated that the *lukSF-PV*^+^ HA isolates might lead to the severity of clinical infections similar to that caused by *lukSF-PV*^+^ CA isolates.

Being a retrospective study, this study had several limitations. First, the corresponding clinical samples could not be collected to evaluate the actual concentration of PVL *in vivo*. Because the PVL production depends on many factors, such as environmental factors, host factors, and internal factors of bacteria, it is not yet known which factor impacts mainly on the protein expression level. Second, the representativeness of isolates from only one hospital was insufficient for determining the generation and isoforms of PVL and the association between PVL production and PVL variants or genetic background or infection types. Third, more clinical information could not be collected to evaluate the correlation of the yield and isoform of PVL with the severity of illness.

In summary, the present experimental data did not sustain the direct correlation of PVL production *in vitro* with clinical types of infection and its isoform. The *in vitro* production of PVL by *lukSF-PV*^+^ MRSA isolates was dramatically changeable, indicating the existence of unknown environmental and host factors in transcriptional and/or translational regulation of gene expression. The regulation mechanisms affecting the differential expression of PVL and the associations among PVL levels and characteristics of isolate and infection require further exploration.

## Methods

### Clinical isolates

Fifty-one consecutive, nonduplicate *lukSF-PV*^+^ MRSA isolates from various clinical specimens of individual patients were obtained from Zhejiang Xiaoshan Hospital (a tertiary hospital), Hangzhou City, China. The isolates were collected between January 2010 and December 2011 and identified using VITEK 32 instrument (bioMérieux, Marcy l′ Etoile, France). Methicillin resistance was determined using the Kirby–Bauer method with cefoxitin disk (30 µg, Oxoid, Basingstoke, UK) and PCR for mecA^33,34^. All isolates had been previously described by PVL isoform detection and PFGE-SCCmec-MLST typing (including CC) (Table 1)^35^. The strains ATCC 49775 and MRSA N315 were used as positive and negative controls for the PVL production, respectively.

### Quantitative detection of PVL

For quantifying the production of PVL *in vitro*, a certain amount of *lukSF-PV*^+^ MRSA (1 to 3 × 10^6^ CFU/mL) was cultured at 37 °C in 5 mL of casein–casein–yeast extract broth in which the optimal production of PVL could be acquired, with shaking at 200 rpm for 20 h. At this time, all growth isolates had reached a survival plateau of 1 to 4 × 10^9^ CFU/mL^6,11^. Culture supernatants were acquired by centrifugation at 8000 *g* for 15 min. The quantification of secreted PVL was performed using ELISA, according to the kit instructions (Shanghai Y-J Biotechnology Co., Ltd., China). Each sample was measured in triplicate. The ELISA detection limit of PVL was 0.3~10 μg/mL. The samples with a concentration of more than 10 μg/mL were diluted for the redetection of PVL. A previous study reported that the intracutaneous injection of 0.3 μg/mL PVL led to local inflammation and necrosis in rabbits, which, like humans, are sensitive to similar PVL concentration^11^. Therefore, a concentration of 0.3 μg/mL was used in the present study, just the lower detection limit of the ELISA kit, as the cutoff value to determine whether the supernatant was PVL positive.

### Definitions

(1) SSTI was described as the existence of a skin abscess or other symptoms of inflammation and a positive MRSA culture^36^. (2) Pneumonia was described as the existence of fever with imaging changes corresponding to pulmonary infection and MRSA-positive respiratory tract specimens (sputum, endotracheal aspirate, bronchial washing, and bronchoalveolar lavage) or blood cultures^36^. (3) Surgical site infection was defined as an infected postoperative wound and drainage with a positive MRSA culture^36^. (4) Primary bacteremia was defined as the existence of a blood culture positive for MRSA without the recognizable origin of infection^36^. (5) Urinary tract infection was defined as MRSA bacteriuria (≥105 CFU/mL) and pyuria. (6) Acute bacterial tracheitis was diagnosed according to the presentation of upper respiratory tract obstruction, such as stridor, cough, and dysphagia; abundant purulent tracheal excretions; and positive culture for MRSA from the tracheal excretions. (7) Tympanitis was defined as the inflammation of the middle ear with a positive MRSA culture. (8) Conjunctivitis was characterized as a positive culture for MRSA plus two or more of the following symptoms: red, watery, itchy eyes; painful, burning eyes; purulent fluid in eyes; or photophobia. (9) CA MRSA infection was described as MRSA infection emerging in the community or within 48 h after admission to hospital without established risk factors (such as hospitalization history, surgery, dialysis, or living in a long-term care residence within 1 year; existence of a long-term indwelling catheter or other percutaneous medical device; or previous MRSA isolation). (10) HA MRSA infection was characterized as strains isolated from patients in less than 48 h of hospitalization with the aforementioned risk factors or >48 h of admission^37^.

### Clinical information

The clinical data of the patients were collected retrospectively from the case notes. The data included gender, age, outpatient or inpatient, clinical symptoms, sample type, date of strain isolation, clinical diagnosis, and other medical histories related to HA or CA infections.

### Statistical analysis

In view of the uneven distribution of PVL production among isolates, the comparisons of two and ≥3 groups were analyzed using the Wilcoxon rank-sum test and the Kruskal–Wallis rank-sum test (SPSS 22.0), respectively. A two-sided *P* value < 0.05 was considered statistically significant.

### Data availability

Most of the experimental data acquired/analyzed during this study have been included in this published version. Information on rest of the data can be obtained from the corresponding author on request.

### Ethics approval and consent to participate

The ethics committee of Zhejiang Xiaoshan Hospital provided ethical approval for this study. The approval ID number was XSYY2015012. All study participants provided an informed consent before participation, and patient information was anonymized.
